# Thinness negatively affects lung function among Sri Lankan children

**DOI:** 10.1371/journal.pone.0272096

**Published:** 2022-08-02

**Authors:** Niroshani Senevirathna, Lakmali Amarasiri, Deepal Jayamanne, Kanthi Manel, Guwani Liyanage

**Affiliations:** 1 Department of Nursing and Midwifery, Faculty of Allied Health Sciences, General Sir John Kotelawala Defence University, Dehiwala-Mount Lavinia, Sri Lanka; 2 Department of Physiology, Faculty of Medicine, University of Colombo, Colombo, Sri Lanka; 3 Department of Public Health, Faculty of Medicine, University of Kelaniya, Colombo, Sri Lanka; 4 Department of Social Statistics, Faculty of Humanities and Social Sciences, University of Sri Jayewardenepura, Nugegoda, Sri Lanka; 5 Department of Paediatrics, Faculty of Medical Sciences, University of Sri Jayewardenepura, Nugegoda, Sri Lanka; Clinic for Infectious and tropical diseases, Clinical centre of Serbia, SERBIA

## Abstract

**Background:**

There have been conflicting findings on the effect of body mass index (BMI) on lung functions in children. Therefore, we studied the relationship between spirometry parameters and BMI among healthy Sri Lankan school children aged 5–7 years.

**Methods:**

A cross-sectional study was conducted among 296 school children (5–7-year-old) without apparent lung disease. Recruitment was done with stratified random sampling. Spirometry parameters, FEV_1_, FVC, PEFR, and FEV_1_/FVC ratio were determined. The acceptable and reproducible spirometry recordings were included in the analysis. Simple and multivariate linear regression analysis examined possible associations of lung function parameters with BMI, socio‐demographic variables and indoor risk factors. Also, the mediator effect of gender on lung function through BMI was explored.

**Results:**

The participants’ mean age (SD) was 6.4 (0.65) years. One-third were thin/severely thin (37%). A statistically significant difference in FVC (p = 0.001) and FEV_1_ (p = 0.001) was observed between BMI groups (obesity/overweight, normal, and thinness). Yet, PEFR or FEV_1_/FVC did not significantly differ among BMI groups (p = 0.23 and p = 0.84). Multivariate regression analysis showed that FEV_1_ and FVC were significantly associated with BMI, child’s age, gender, family income, father’s education, having a pet, and exposure to mosquito coil smoke. Interaction between gender and BMI for lung functions was not significant. The thin children had significantly lower FVC (OR: -0.04, 95%CI: -0.077, -0.012, p = 0.008) and FEV_1_ (OR: -0.04, 95%CI: -0.075, -0.014, p = 0.004) than normal/overweight/obese children. Family income demonstrated the greatest effect on lung functions; FVC and FEV_1_ were 0.25L and 0.23L smaller in low-income than the high-income families.

**Conclusion:**

Lower lung function parameters (FVC and FEV_1_) are associated with thinness than normal/overweight/obese dimensions among children without apparent lung disease. It informs that appropriate nutritional intervention may play a role in improving respiratory health.

## Introduction

Undernutrition is a major health concern in low-and middle-income countries. In these same countries, childhood overweight and obesity rates are also rising [1]. Naotunna et al., reported 33.5% and 4.5% of thinness (BMI z-score for age <-2SD) and overweight/obesity (BMI z-score for age >+1SD) [2, 3] in 6-7-year-old Sri Lankan children.

Plentiful scientific evidence supports the connections between obesity and respiratory and non-respiratory disorders [4, 5]. Low weight, too, is associated with increased respiratory morbidity [6, 7]. Due to respiratory tract infections, the risk of being hospitalized is more in underweight or morbidly obese individuals [8]. Many studies have explored the connection between obesity and lung function; however, very few have reported the impact of thinness on lung function [9–14]. Further, the findings of those studies are inconsistent, probably due to varying study designs, geographic locations, and socio-cultural differences among the study participants. Also, not many studies have investigated determinants of lung function in pre-pubertal children [12]. Therefore, the connection between low BMI and lung function in young children remains poorly understood. In the Sri Lankan context, the effect of BMI and other factors (viz. sociodemographic, indoor risk factors) on lung function has not been studied in children less than eight years [14]. We hypothesized that BMI affects lung function in children. Therefore, our main objective was to study the relationship between spirometry parameters and BMI among healthy Sri Lankan school children aged 5–7 years.

## Materials and methods

### Study design, setting, and participants

A cross-sectional study was conducted from February to August 2019, involving healthy school children aged 5 to 7 years in 15 schools in the Colombo district. A two-stage stratified sampling technique was used. The minimum sample size was 290. The expected proportion of children with acceptable spirometry curves for sample calculation was 78% [15]. Also, a level of precision of 0.05, a confidence interval of 0.05, and a non-response rate of 10% were considered in the calculation. Then, stratified by age and gender, one class from each grade was randomly selected from each school (proportionate to size). Both children and parents were informed about the study procedure, and the written consent of parents was obtained. Children with acute respiratory symptoms at the time of sample collection or in the previous two weeks, chronic respiratory disorders, neurological disorders, or other significant chronic illnesses were excluded from the study.

### Procedure

The parents/caregivers completed a pre-tested, self-administered questionnaire that gathered socio-demographic details (age, gender, mother’s and father’s educational attainment, and household income), indoor risk factors (cigarette smoke, mosquito coils, having pets) and general health, including respiratory health (S1 File). After that, all children underwent physical examination and spirometry measurements at the school premises. Height was measured using a stadiometer (Seca GmbH & Co. KG), and the measurements were taken to the nearest 0.1 cm without shoes or socks. Weight measured in kilograms and grams to the nearest 100g electronic flat weighing scale (Seca GmbH & Co. KG), wearing lightweight clothing. The BMI was calculated as [weight (kg)/height (m^2^)] and categorized according to the World Health Organization Growth Standards [Z scores for obesity >+2SD, overweight +2SD to >+1SD, Normal +1SD to -2SD, thinness <-2SD] [3].

The spirometry procedure followed the American Thoracic Society (ATS) guidelines [16] using a microQuark PC-based spirometer (COSMED, Italy). The spirometry procedure was conducted by the same investigator (NS). A bacterial-viral filter and a disposable mouthpiece were used for each child. The following parameters were assessed; forced vital capacity (FVC), forced expiratory volume in the first second (FEV1), peak expiratory flow rate (PEFR), and FEV_1_/FVC ratio. The machines were calibrated using a standard three-liter syringe at the examination site each day prior to taking measurements. All children were given clear instructions prior to testing. A nose clip was used to occlude the nostrils. They were all tested in the standing position. The children were asked to take tidal breaths initially, then take a deep breath filling the chest, and blow out fast, hard, and as long as possible. An animation program on the computer screen was used to encourage the children. A maximum of five attempts was allowed. They were allowed to discontinue more than five attempts or earlier if the child was not interested in continuing. All measurements were performed by one investigator using the same equipment. Two authors of this study analyzed the tests for acceptability and reproducibility (LA and GL).

### Statistical analysis

Statistical analysis was done using SPSS version 22. Extreme outliers were identified, and data were checked for normality. The extreme outliers in lung function variables (FEV1, FVC, PEF, weight, and BMI) due to transcription errors that could not be resolved were removed. Descriptive statistics were expressed as mean (standard deviation) and percentages where appropriate. Pearson correlation was used to assess the correlation between BMI (as a continuous variable) and lung function test parameters. Associations of BMI groups (thinness, normal, and overweight/obese) with lung function were performed using one-way ANOVA and the Tukey test. Multivariate regression assessed the association between lung function parameters and BMI groups (thinness vs. normal/overweight/obese) as the predictor variable and other confounding variables (socio-demographic, indoor risk factors, etc.). Before multivariate regression analysis, variables with high multicollinearity were excluded (viz., mother’s education). Residual and scatter plots indicated the assumptions of normality and homoscedasticity.

### Ethical consideration

Ethics approval was obtained from the Ethics Review Committee of the Faculty of Medical Sciences, University of Sri Jayewardenepura. The study was carried out following the guidelines of the Declaration of Helsinki. Informed written consent was taken from the parent/guardian before data collection.

## Results

A total of 357 children were invited. Two hundred ninety-six technically acceptable flow-volume curves were included in the analysis. In 46 children, the spirometry curves did not comply with ATS standards. There were 15 incomplete questionnaires. The response rate was 83%. Basic characteristics of the sample, including anthropometry and spirometry parameters, are shown in Table 1. The participants’ mean age (SD) was 6.4 (0.65) years. Most were males (63.2%). Almost half (44%) were in the higher income category. More than two-thirds of the parents had either secondary or post-secondary education. Most children had normal BMI (55%), while one-third had thinness (37%).

**Table 1:** Basic characteristics of the participants.

	n	%
Age (years), mean (SD)	6.4 (0.65)	-
Sex (males)	187	63.2
Birthweight (kg), mean (SD)	2.93	-
Mother’s education		
Less than secondary	67	22.6
Secondary	115	38.9
Post-secondary	113	38.5
Father’s education		
Less than secondary	65	22
Secondary	123	41.6
Post-secondary	108	36.5
*Family income (LKR)		
Low	166	56.1
High	130	43.9
Having a pet	78	26.4
Exposure to passive smoking	41	13.9
Using mosquito coils	112	37.8
Family history of atopy	104	35.1
Anthropometry		
Height (m), mean (SD)	1.17 (0.1)	-
Weight (Kg), mean (SD)	18.96 (3.3)	-
BMI (kg/m2), mean (SD)	13.85 (1.80)	-
BMI category Thinness	109	36.8
Normal	163	55
Overweight/obese	24	8.2
Spirometry parameters		
FEV_1_ (L), mean (SD)	0.99 (0.2)	-
FVC (L), mean (SD)	1.12 (0.2)	-
PEFR (L/s), mean (SD)	2.54 (0.6)	-
FEV_1_/FVC, mean (SD)	0.89 (0.23)	-
FEV_1_/FVC% >70%	296	100

Expressed as n and %, unless otherwise indicated.

*Low income <60,000 LKR and high income ≥60,000 LKR.

Weak but significant positive correlations were found between BMI and FVC (r = 0.22, p<0.001). A similar association was noted with FEV_1_ (r = 0.23, p<0.001) and PEFR (r = 0.15, p = 0.02). FEV_1_/FVC ratio did not show a significant association with BMI (p = 0.19). Lung function parameters were positively correlated with age FVC (r = 0.31, p<0.001), FEV_1_ (r = 0.33, p<0.001), PEFR (r = 0.30, p<0.001), and FEV_1_/FVC ratio (r = 0.23, p<0.001). As determined by one-way ANOVA, there was a statistically significant difference in FVC (p-0.001) and FEV_1_ (p = 0.001) between BMI groups (obesity/overweight, normal, and thinness) (Table 2). Yet, PEFR and FEV_1_/FVC were not significantly associated with BMI groups (p = 0.23 and p = 0.84, respectively).

**Table 2:** Relationship between spirometry parameters and BMI groups.

	BMI category			P-value
	Thin/severely thin	Normal	Obese/ overweight	
FEV_1_ (L)	0.95 (0.20)	1.02 (0.19)	1.08 (0.26)	0.001
FVC (L)	1.07 (0.21)	1.14 (0.20)	1.20 (0.27)	0.001
PEFR (L/s)	2.46 (0.57)	2.58 (0.60)	2.62 (0.73)	0.23
FEV_1_/FVC	0.90 (0.02)	0.90 (0.02)	0.89 (0.03)	0.84

Comparisons using One-way ANOVA.

Abbreviations: BMI-Body mass index, FEV_1_-Forced expiratory volume in the first second, FVC-Forced vital capacity.

Tukey’s post hoc test revealed that FVC was significantly low in the thinness group compared to the normal BMI (p = 0.02) and obese/overweight groups (p = 0.002). There was no statistically significant difference in FVC between the normal BMI group and the overweight/obese group (p = 0.10). Likewise, FEV_1_ was significantly low in the thinness group compared to the normal BMI (p = 0.04) and obese/overweight (p = 0.001) groups. There was no statistically significant difference in FEV_1_ between the normal BMI group and the overweight/obese group (p = 0.06). PEFR was not different between the thinness group and normal (p = 0.25) or obese/overweight groups (p = 0.51). Also, no difference was found in PEFR between the normal and obese/overweight groups (p = 0.97). FEV_1_/FVC was not significantly different between thinness and overweight/obese (p = 0.88) or normal groups (p = 0.87). Similarly, there was no significant difference in FEV_1_/FVC between normal and overweight/obese groups (p = 0.97).

Further analysis using linear regression was done to evaluate the effect of being thin on lung function parameters. Thus, subjects were divided into thinness and otherwise (normal/overweight/obese) groups. Univariate analysis of independent variables, including BMI, is given in the S1 File. All significant variables were considered in the multiple linear regression (age, gender, birth weight, mother’s education, father’s education, income, having pets, exposure to passive smoking, exposure to mosquito coils, and family history of atopy). However, the mother’s education, exposure to passive smoking, and birth weight were not included as they did not improve the model significantly. The interaction effect of gender and BMI were considered in the regression model; however, it was not included in the final model as it did not significantly impact FVC or FEV_1_. The multivariate regression statistics are shown in Table 3. The thinness group had lower FEV_1_ and FVC than the normal/overweight/obese group. In children from low-income families, FVC and FEV_1_ were 0.25L and 0.23L lower than those from high-income families. Similarly, age, gender, father’s education, having pets, and exposure to mosquito coils were independently associated with lung function parameters.

**Table 3:** Multivariate regression analysis for predictors of FEV_1_ and FVC.

	FVC				FEV_1_			
	B	95% CI for B		P value	B	95% CI for B		P value
Age	0.01	0.003	0.007	<0.001	0.01	0.004	0.008	<0.001
Gender (female)	-0.06	-0.090	-0.024	0.001	-0.06	-0.090	-0.028	<0.001
Father’s education								
Secondary/post- secondary	1 (ref)							
Less than secondary	-0.04	-0.080	-0.002	0.04	-0.04	-0.080	-0.008	0.02
Income								
High	1 (ref)							
Low	-0.25	-0.287	-0.214	<0.001	-0.23	-0.259	-0.192	<0.001
BMI								
Normal/overweight/obese	1 (ref)							
Thinness	-0.04	-0.077	-0.012	0.008	-0.05	-0.075	-0.014	0.004
Mosquito coil smoke (exposed)	-0.05	-0.084	-0.018	0.003	-0.05	-0.080	-0.019	0.002
Having pets	-0.04	-0.079	-0.008	0.02	-0.04	-0.075	-0.009	0.01

Model for FVC: F 6,283 = 71.7; p<0.001; R^2^ = 0.61, Model for FEV1: F 6,282 = 68.9; p<0.001; R^2^ = 0.60. Abbreviations: BMI-Body mass index, CI-Confidence interval, FEV1-Forced expiratory volume in the first second, FVC: Forced vital capacity.

## Discussion

In this cross-sectional study of 5-7-year-old children without apparent lung disease, thinness was independently associated with reduced FVC and FEV_1_. Comparably, a retrospective study of 327 children reported a lower percent predicted FVC and vital capacity (VC) in the underweight group (BMI <5th percentile) compared to the obese group [12]. Another study among Korean adults reported decreased lung functions (FEV_1_ and FVC) with low BMI (<18.5kg/m2) [9]. In thin individuals, reduced lung functions may be attributed to low muscle mass in the abdomen, diaphragm, and chest wall [17]. Supporting this notion, a previous study among Korean adults reported low FVC and FEV_1_ among thin individuals with reduced muscle mass [18].

We observed that overweight/obese children had higher FVC and FEV_1_ than normal-weight children, even though the difference was insignificant. In a previous Sri Lankan study of 8–16 years old, children with obesity/overweight had significantly higher lung functions (FVC, FEV1, and PEFR) than normal-weight children [14]. Nevertheless, the opposite had been described in several other studies, in which obesity negatively affected lung functions [12, 19]. Essentially, BMI indicates weight in relation to height. It does not distinguish fat mass from fat-free mass [20]. Therefore, body composition may vary among populations with the same BMI [21]. It explains the basis for the differences between the above studies as variable lung functions (FVC and FEV1) are expected with different body compositions.

As expected, the gender difference in FVC and FEV1 were observed. However, a BMI-gender interaction on lung functions was not observed. Previous studies have reported different results. Tantisira et al. observed a stronger interaction between BMI and lung function in girls compared to boys [22]. It was readily explained by differential airways size. Girls have larger airways in relation to lung size throughout childhood than boys, a phenomenon that begins to reverse in adolescence [23]. The exact reasons for the absence of BMI-gender interaction in our cohort cannot be clearly explained.

Indoor risk factors such as exposure to passive smoking and mosquito coils and having a pet were negatively associated with FEV1 and FVC. In a Mexican study, FEV1 and FVC in children between 8–17 years exposed to passive smoking were 6.8 and 14.1 ml lower than those of non-exposed children [24]. These values decreased with increasing smokers at home [24]. In Asian countries, burning mosquito coils is a common indoor environmental pollutant [25]. It releases continuous smoke, and the particle amounts and sizes of the smoke are comparable to other indoor sources of smoke [25]. Liu et al. reported that burning one mosquito coil would produce the same particulate amount of matter as burning 75–137 cigarettes [26]. Animals are the third leading cause of allergic asthma, after mites and pollens [27]. Therefore, it is likely that having a pet may impact lung functions.

Socioeconomic indices (income and educational level) were significant predictors of lung functions. Largely, low SES is linked to poor housing, inadequate ventilation, and adult smokers [28, 29]. Exposure to those risk factors and increased airway inflammation among low SES would explain the reduced lung functions.

In the present study, PEFR had a weak correlation with BMI. Different studies have reported varying associations between BMI and PEFR; many have reported a reduction in PEFR in obesity [9]. The interplay between many airway factors (airway size), effort, BMI, and age is thought to be very complex; thus, it is not surprising to have varying results [30]. The difference in mean FEV_1_/FVC was insignificant between the BMI groups. The lack of association is probably due to each parameter (viz. FVC and FEV1) getting affected at a similar rate so that the ratio remains unaffected. Yet, several other studies have observed a significant negative relationship with low FEV1/FVC ratio among obese subjects [11, 12, 19].

The findings of this study should be interpreted with the following limitations. First, a longitudinal study would have evaluated the lung function with changes in BMI over a period than a one-time cross-sectional assessment. Secondly, not considering the cluster effect in the sample size calculation was a limitation. Thirdly, we could not gather data on geographical area, vaccination status, and gestational age at birth. Furthermore, since recurrent respiratory tract infections were an exclusion criterion, we could not assess the relationship between recurrent respiratory tract infections and BMI. In recurrent respiratory infections, low lung functions could be related to malnutrition. Finally, we evaluated the body composition with BMI, not the muscle mass or fat-free mass. Therefore, further studies are particularly important to assess the impact of muscle mass on lung function in resource-poor settings. Finally, despite all these limitations, including a healthy population of 5–7-year-old children, screening through a well-designed questionnaire and a clinical examination was a strength of our study.

## Conclusions

Lower lung functions (FVC and FEV1) are associated with thinness more than normal/overweight/obese dimensions among children without apparent lung disease. Thus, it informs that appropriate nutritional intervention may play a role in improving respiratory health. Further, this study emphasizes the relationship of selected sociodemographic and indoor risk factors on lung function parameters in this population. Further investigation of other potential factors such as recurrent respiratory infections and genetic composition will help better understand lung function associated with thinness.

## Supporting information

S1 File Contains a supporting table and the study questionnaire. (DOCX)

## References

[1] World Health Organisation. Malnutrition. 9 June 2021. Available at https://www.who.int/news-room/fact-sheets/detail/malnutrition

[2] Naotunna NP, Dayarathna M, Maheshi H, Amarasinghe GS, Kithmini VS, Rathnayaka M, et al. Nutritional status among primary school children in rural Sri Lanka; a public health challenge for a country with high child health standards. BMC Public Health. 2017 Jan 10;17(1):57. doi: 10.1186/s12889-016-4001-1 28068960 PMC5223320

[3] World Health Organisation. BMI for age (5–19 years). Available at https://www.who.int/tools/growth-reference-data-for-5to19-years/indicators/bmi-for-age

[4] Safaei M, Sundararajan EA, Driss M, Boulila W, Shapi’i A. A systematic literature review on obesity: Understanding the causes & consequences of obesity and reviewing various machine learning approaches used to predict obesity. Comput Biol Med. 2021 Sep;136:104754. doi: 10.1016/j.compbiomed.2021.104754 34426171

[5] Mafort TT, Rufino R, Costa CH, Lopes AJ. Obesity: systemic and pulmonary complications, biochemical abnormalities, and impairment of lung function. Multidiscip Respir Med. 2016 Jul 12;11:28. doi: 10.1186/s40248-016-0066-z 27408717 PMC4940831

[6] Bejon P, Mohammed S, Mwangi I, Atkinson SH, Osier F, Peshu N, et al. Fraction of all hospital admissions and deaths attributable to malnutrition among children in rural Kenya. Am J Clin Nutr. 2008 Dec;88(6):1626–31. doi: 10.3945/ajcn.2008.26510 19064524 PMC2635111

[7] Furukawa T, Hasegawa T, Suzuki K, Koya T, Sakagami T, Youkou A, et al. Influence of underweight on asthma control. Allergol Int. 2012 Sep;61(3):489–96. doi: 10.2332/allergolint.12-OA-0425 22824977

[8] Moser JS, Galindo-Fraga A, Ortiz-Hernández AA, Gu W, Hunsberger S, Galán-Herrera JF, et al. La Red ILI 002 Study Group. Underweight, overweight, and obesity as independent risk factors for hospitalization in adults and children from influenza and other respiratory viruses. Influenza Other Respir Viruses. 2019 Jan;13(1):3–9. doi: 10.1111/irv.12618 30515985 PMC6304312

[9] Do JG, Park CH, Lee YT, Yoon KJ. Association between underweight and pulmonary function in 282,135 healthy adults: A cross-sectional study in Korean population. 2019;Sci Rep 9, 14308. doi: 10.1038/s41598-019-50488-3 31586079 PMC6778122

[10] Ferreira MS, Marson FAL, Wolf VLW, Ribeiro JD, Mendes RT. Lung function in obese children and adolescents without respiratory disease: a systematic review. BMC Pulm Med. 2020;20(1):281. doi: 10.1186/s12890-020-01306-4 33115462 PMC7594270

[11] Forno E, Han YY, Mullen J, Celedón JC. Overweight, Obesity, and Lung Function in Children and Adults-A Meta-analysis. J Allergy Clin Immunol Pract. 2018 Mar-Apr;6(2):570–581.e10. doi: 10.1016/j.jaip.2017.07.010 28967546 PMC5845780

[12] Davidson WJ, Mackenzie-Rife KA, Witmans MB, Montgomery MD, Ball GD, Egbogah S, et al. Obesity negatively impacts lung function in children and adolescents. Pediatr Pulmonol. 2014 Oct;49(10):1003–10. doi: 10.1002/ppul.22915 24167154

[13] Dong Y, Lau PWC, Dong B, Zou Z, Yang Y, Wen B, et al. Trends in physical fitness, growth, and nutritional status of Chinese children and adolescents: a retrospective analysis of 1·5 million students from six successive national surveys between 1985 and 2014. Lancet Child Adolesc Health. 2019 Dec;3(12):871–880. doi: 10.1016/S2352-4642(19)30302-5 31582349

[14] Liyanage G, Jayamanne B D, Aaqiff M, Sriwardhana D. Effect of body mass index on pulmonary function in children. Ceylon Med J. 2016 Dec 30;61(4):163–166. doi: 10.4038/cmj.v61i4.8382 28076945

[15] Nystad W, Samuelsen SO, Nafstad P, Edvardsen E, Stensrud T, Jaakkola JJ. Feasibility of measuring lung function in preschool children. Thorax. 2002;57(12):1021–1027. doi: 10.1136/thorax.57.12.1021 12454295 PMC1758804

[16] Graham BL, Steenbruggen I, Miller MR, et al. Standardization of Spirometry 2019 Update. An Official American Thoracic Society and European Respiratory Society Technical Statement. Am J Respir Crit Care Med. 2019;200(8):e70–e88. doi: 10.1164/rccm.201908-1590ST 31613151 PMC6794117

[17] Jensen ME, Gibson PG, Collins CE, Wood LG. Lean mass, not fat mass, is associated with lung function in male and female children with asthma. Pediatr Res. 2014 Jan;75(1–1):93–8. doi: 10.1038/pr.2013.181 24129552

[18] Park CH, Yi Y, Do JG, Lee YT, Yoon KJ. Relationship between skeletal muscle mass and lung function in Korean adults without clinically apparent lung disease. Medicine (Baltimore). 2018 Sep;97(37):e12281. doi: 10.1097/MD.0000000000012281 30212965 PMC6155967

[19] Spathopoulos D, Paraskakis E, Trypsianis G, Tsalkidis A, Arvanitidou V, Emporiadou M, et al. The effect of obesity on pulmonary lung function of school aged children in Greece. Pediatr Pulmonol. 2009 Mar;44(3):273–80. doi: 10.1002/ppul.20995 19208374

[20] Chung S. Body mass index and body composition scaling to height in children and adolescent. Ann Pediatr Endocrinol Metab. 2015;20(3):125. doi: 10.6065/apem.2015.20.3.125 26512347 PMC4623339

[21] Weber DR, Leonard MB, Zemel BS. Body composition analysis in the pediatric population. Pediatr Endocrinol Rev. 2012;10(1):130–139. 23469390 PMC4154503

[22] Tantisira KG, Litonjua AA, Weiss ST, Fuhlbrigge AL. Childhood Asthma Management Program Research Group. Association of body mass with pulmonary function in the Childhood Asthma Management Program (CAMP). Thorax. 2003 Dec;58(12):1036–41. doi: 10.1136/thorax.58.12.1036 14645968 PMC1746552

[23] Becklake MR, Kauffmann F. Gender differences in airway behaviour over the human life span. Thorax 1999; 54: 1119–1138. doi: 10.1136/thx.54.12.1119 10567633 PMC1763756

[24] Fernández-Plata R, Rojas-Martínez R, Martínez-Briseño D, García-Sancho C, Pérez-Padilla R. Effect of Passive Smoking on the Growth of Pulmonary Function and Respiratory Symptoms in Schoolchildren. Rev Invest Clin. 2016 May-Jun;68(3):119–27. 27408998

[25] Lin TS, Shen FM. Trace metals in mosquito coil smoke. Bull Environ Contam Toxicol. 2005 Jan;74(1):184–9. doi: 10.1007/s00128-004-0566-y 15768517

[26] Liu W, Zhang J, Hashim JH, Jalaludin J, Hashim Z, Goldstein BD. Mosquito coil emissions and health implications. Environ Health Perspect. 2003 Sep;111(12):1454–60. doi: 10.1289/ehp.6286 12948883 PMC1241646

[27] González-Mancebo E, Martín-García C, Núñez-Acevedo B, Prior N, Reche M, Rosado A, et al. Consensus document on dog and cat allergy. Allergy. 2018 Jun;73(6):1206–1222. doi: 10.1111/all.13391 29318625

[28] Gaffney AW, Hang JQ, Lee MS, Su L, Zhang FY, Christiani DC. Socioeconomic status is associated with reduced lung function in China: an analysis from a large cross-sectional study in Shanghai. BMC Public Health. 2016;16:96. Published 2016 Feb 1. doi: 10.1186/s12889-016-2752-3 26832923 PMC4736183

[29] Oguonu T, Obumneme-Anyim IN, Eze JN, Ayuk AC, Okoli CV, Ndu IK. Prevalence and determinants of airflow limitation in urban and rural children exposed to cooking fuels in South-East Nigeria. Paediatr Int Child Health. 2018;38(2):121–127. doi: 10.1080/20469047.2018.1445506 29542392

[30] Salome CM, King GG, Berend N. Physiology of obesity and effects on lung function. Journal of Applied Physiology 2006;108: 11. doi: 10.1152/japplphysiol.00694.2009 19875713

