# Supplementary material for: Thinness negatively affects lung function among Sri Lankan children
Source: PLoS One. 2022 Aug 2;17(8):e0272096. doi: 10.1371/journal.pone.0272096 (PMC9345351; doi:10.1371/journal.pone.0272096)
Supplement: S1 File — (DOCX) [file pone.0272096.s001.docx]

Univariate analysis of predictors of FVC and FEV1

|  | FVC | | | | | FEV_1_ | | | |
| --- | --- | --- | --- | --- | --- | --- | --- | --- | --- |
|  | B | CI for B | | | P value | B | CI for B | | P value |
| Age | 0.01 | 0.006 | | 0.012 | <0.001 | 0.01 | 0.007 | 0.012 | <0.001 |
| Gender (female) | -0.13 | -0.176 | | -0.077 | <0.001 | -0.12 | -0.170 | -0.077 | <0.001 |
| Birth weight | 0.12 | 0.43 | | 0.198 | 0.002 | 0.11 | 0.042 | 0.188 | 0.002 |
| Education (Father) |  |  | |  |  |  |  |  |  |
| Secondary/post-secondary | 1 (ref) | | | | | 1 (ref) | | | |
| Less than secondary | -0.15 | -0.209 | | -0.095 | <0.001 | -0.15 | -0.200 | -0.093 | <0.001 |
| Education (Mother) |  |  | |  |  |  |  |  |  |
| Secondary/post-secondary |  |  | |  |  |  |  |  |  |
| Less than secondary | -0.15 | 0.209 | | 0.097 | <0.001 | -0.014 | -0.191 | 0.085 | <0.001 |
| Income |  |  | |  |  |  |  |  |  |
| High | 1 (ref) | |  | | | 1 (ref) | | | |
| Low | -0.32 | -0.354 | | -0.285 | <0.001 | -0.30 | -0.330 | -0.265 | <0.001 |
| Family history of atopy | 0.11 | 0.062 | | 0.162 | <0.001 | 0.11 | 0.059 | 0.154 | <0.001 |
| Passive smoking | -0.07 | -0.142 | | -0.002 | 0.05 | -0.07 | -0.139 | -0.006 | 0.03 |
| Having pets | -0.11 | -0.162 | | -0.053 | <0.001 | -0.10 | -0.154 | -0.051 | <0.001 |
| Mosquito coil smoke (exposed) | -0.14 | -0.185 | | -0.088 | <0.001 | -0.13 | -0.174 | -0.083 | <0.001 |
| BMI |  |  | |  |  |  |  |  |  |
| Normal/overweight/obese | 1 (ref) | |  | | | 1 (ref) | | | |
| Thinness | -0.08 | -0.131 | | -0.029 | 0.002 | -0.08 | -0.126 | -0.030 | 0.002 |
| Abbreviations: BMI-Body mass index, CI-Confidence interval, FEV_1_-Forced expiratory volume in first second, FVC: Forced vital capacity. | | | | | | | | | |

**Questionnaire**

Spirometry on 5–7-year-old children in the Colombo District

**Part A: Screening questionnaire**

1. Has your child had a dry cough at night apart from cough and cold associated with chest infections in the past 12 months? Yes/ No
2. Has your child had a wheezing, whistling or continuous cough at night in the past 12 months? Yes/ No
3. Has your child’s chest sounded wheezy, chest tiredness, difficulty in breathing or cough during or after playing or exercises? Yes/ No
4. Is your child often treated with antibiotics for recurrent respiratory tract diseases in the past 12 months? Yes/ No
5. Has your child ever been treated for asthma/recurrent wheezing? Yes/No

If yes, specify………………………………

1. Has your child been treated with ay prolonged medications for any illness? Yes/No

If yes, specify……………………………….

Eligible: Yes/ No

**Part B: Socio-demographic data**

1. Serial no: ……….
2. Grama Niladari division: …………………………….
3. Date of birth: ……………. Age: Years ……. Months: …………
4. What is your child’s birth weight? .............................................
5. Sex: Male/ Female
6. Name of the school: …………………………………………. Class…………………….
7. Ethnicity……………….
8. Interviewee relation to the child: Mother/Father/Legal guardian: …………………………
9. Educational status of mother: not schooled/ primary/ secondary school/diploma or certificate course/degree/postgraduate: …………………………………………
10. Educational status of father: not schooled/ primary/ secondary school/diploma or certificate course/degree/postgraduate: ……………………………
11. Does any of your family members (mother / father / siblings/ Grandmother /Grandfather) have wheezing/asthma/ catarrh/ eczema? Yes/ no
12. If yes, what? ………………………………………………………………………………….

**Part C : Indoor risk factors**

1. Does your child get exposed to mosquito coils? Yes / No
2. Does anyone smoke cigarettes inside the house? Yes / No
3. Did you have a pet during the last 12 months? Yes / No:

If yes, what is your pet: cat: dog: other:

1. Is your house close to the road? Yes / No
2. Have you got factories/workplaces in and around your house which gives fumes and chemicals? Yes / No
3. How many rooms are there in your house? …………….

**Part D: Physical examination**

1. Height:
2. Weight:
3. BMI:
4. General Examination:
5. Respiratory tract Examination:

**Part E: Spirometry findings**

1. Acceptability:
2. Reproducibility:

|  | Predicted value | Measured value | % predicted |
| --- | --- | --- | --- |
| 1. FEV1: |  |  |  |
| 1. FVC: |  |  |  |
| 1. FEV1/FVC: |  |  |  |
| 1. PEF: |  |  |  |

1. Overall comment:
